# 2-Arylidene-1-indandiones as Pleiotropic Agents with Antioxidant and Inhibitory Enzymes Activities †

**DOI:** 10.3390/molecules24234411

**Published:** 2019-12-03

**Authors:** Olympia Kouzi, Eleni Pontiki, Dimitra Hadjipavlou-Litina

**Affiliations:** Department of Pharmaceutical Chemistry, School of Pharmacy, Faculty of Health Sciences, Aristotle University of Thessaloniki, 54124 Thessaloniki, Greece; kpaulina2000@yahoo.com (O.K.); epontiki@pharm.auth.gr (E.P.)

**Keywords:** 2-arylidene-1,3-indandiones, lipoxygenase inhibitors, antioxidants, Lipinski “Rule of five”

## Abstract

Indandiones are a relatively new group of compounds presenting a wide range of biological activities. The synthesis of these compounds was performed via a Knoevenagel reaction between an aldehyde and 1,3-indandione and were obtained with a yield up to 54%. IR, ^1^H-Nucleic Magnetic Resonance (NMR), ^13^C-NMR, LC/MS ESI^+^ and elemental analysis were used for the confirmation of the structures of the novel derivatives. Lipophilicity values of compounds were calculated theoretically and experimentally by reversed chromatography method as values R_M_. The novel derivatives were studied through in vitro and in vivo experiments for their activity as anti-inflammatory and antioxidant agents and as inhibitors of lipoxygenase, trypsin, and thrombin. The inhibition of the carrageenin-induced paw edema (CPE) was also determined for representative structures. In the above series of experiments, we find that all the compounds showed moderate to satisfying interaction with the stable DPPH free radical in relation to the concentration and the time 2-arylidene-1-indandione (**10**) was the strongest. We observed moderate or very low antioxidant activities for selected compounds in the decolorization assay with ABTS^+•^. Most of the compounds showed high anti-lipid peroxidation of linoleic acid induced by AAPH.2-arylidene-1-indandione (**7**) showed a strongly inhibited soybean LOX. Only 2-arylidene-1-indandione (**3**) showed moderate scavenging activity of superoxide anion, whereas 2-arylidene-1-indandione (**8**) and 2-arylidene-1-indandione (**9**) showed very strong inhibition on proteolysis. 2-arylidene-1-indandione (**8**) highly inhibited serine protease thrombin. 2-arylidene-1-indandiones (**7**, **8** and **9**) can be used as lead multifunctional molecules. The compounds were active for the inhibition of the CPE (30–57%) with 2-arylidene-1-indandione (**1**) being the most potent (57%). According to the predicted results a great number of the derivatives can cross the Blood–Brain Barrier (BBB), act in CNS and easily transported, diffused, and absorbed. Efforts are conducted a) to correlate quantitatively the in vitro/in vivo results with the most important physicochemical properties of the structural components of the molecules and b) to clarify the correlation of actions among them to propose a possible mechanism of action. Hydration energy as E_HYDR_ and highest occupied molecular orbital (HOMO) better describe their antioxidant profile whereas the lipophilicity as R_M_ values governs the in vivo anti-inflammatory activity. Docking studies are performed and showed that soybean LOX oxidation was prevented by blocking into the hydrophobic domain the substrates to the active site.

## 1. Introduction

Inflammation is a complex protective biological response to injury or infection, representing one of the most important defense mechanisms of an organism to pathogens, damaged cells, or irritants. Inflammatory responses are mainly associated with arachidonic acid (AA-cis 5, 8,11,14-eicosatetraenoic acid)cascade [1]. AA is a twenty-carbon polyunsaturated fatty acid released in response to various stimuli [2], by the activation of phospholipase A_2_ (PLA_2_, generally cytoplasmicPLA_2_) and other lipases [3]. AA once released from the phospholipid bilayer can be further metabolized via three major enzymatic routes: cyclooxygenase (COX), lipoxygenase (LOX) and monooxygenase cytochrome P450 (CYP) to eicosanoids [4]. The COX pathway leads to prostaglandins (PGs), thromboxane and other prostanoids; the 5-, 12- and 15-LOX pathways leads to 5-, 12-, or 15-hydro(-pero)xy-eicosa-tetra-enoic acids [H(P)ETEs] and additionally leukotrienes as well as lipoxins (LXs); and finally the cytochrome P450 monooxygenase pathway leads to diverse epoxy-eicosatetraenoic acids (EETs) and diHETEs [5]. Inflammation is associated with many diseases such atherosclerosis, cardiovascular disorders, neurodegeneration, osteoarthritis, rheumatoid arthritis, diabetes, allergy, infection and cancer [6].

Recent studies have established the implication of inflammatory processes in cardiovascular diseases (initiation, progression and thrombotic complications), formerly considered a bland lipid storage disease [7]. Coronary heart disease (CHD) is characterized as a chronic low-grade inflammatory process. In physiological conditions vascular endothelium regulates homeostasis through the regulation of vascular dilatation, prevention of platelet adhesion, and inhibition of thrombin generation. Endotheliun dysfunction caused by chronic inflammation (atherothrombosis, atherosclerosis, diabetes, or chronic arterial hypertension) or acute inflammation (physical injury of the vascular wall e.g., balloon angioplasty) generates a pro-inflammatory environment supporting leukocyte transmigration toward inflammatory sites [8]. By this state there is enhanced expression of growth factors, adhesion, and signaling molecules, lipid mediators, cytokines, and chemokines controlling leukocytes playing pivotal role in inflammatory response [9]. At the same time, thrombin is realized from the damaged endothelium as a pro-inflammatory mediator provoking fibrin deposition and coagulation [10]. Thrombin, a serine protease plays crucial role in clotting and inflammatory process [8]. During this inflammatory process free radicals are produced from diet-derived lipids in the arterial wall and serum producing peroxides and other reactive oxygen species [11].

The implication of reactive oxygen species (ROS) in disorders associated with oxidative stress (e.g., coronary artery disease, inflammatory injury, cancer and cardiovascular diseases) was acknowledged by various research groups [12,13,14,15,16]. It is well known that ROS e.g., hydroxyl radical, superoxide anion and hydrogen peroxide act by attacking the macromolecules of the organism (proteins, lipids and DNA) [17] provoking chemoresistance, proliferation, genetic instability, radio-resistance, angiogenesis, and invasion [18].

Over the last decade the concept of “promiscuous” medicine has been primarily associated with the capability to selectively attack multiple factors and to address multifactorial diseases. The multi-target concept has been extensively reviewed due to the complex and deleterious consequences of ROS in diverse diseases (atherosclerosis, inflammatory injury, cancer and cardiovascular diseases) implicating inflammatory processes [19]. Combination of more than one activity in one molecule offers an advantage for the therapy of the above-mentioned pathophysiological diseases.

One of the main research interests in our group has been the design of small multi-target molecules with biological activity based on the α, β-unsaturated keto scaffold e.g., chalcones and cinnamic acid derivatives [20,21]. Chalcones are small molecules, known as α, β-unsaturated ketones, presenting exceptional biological interest [22]. The medicinal significance of the enone moiety and of chalcones is highlighted by numerous publications [23,24,25,26,27]. The bioactivity of chalcones depends on the conjugated double bond with the carbonyl moiety, as removal of the enone group renders them inactive. Indandiones are classified as bicyclic aromatic β-diketones. They have been predominantly applied as substrates in organic and medicinal chemistry as their derivatives have interesting biological activities such as: antitumor, antiplatelet, anti-inflammatory, antimicrobial, neuroprotective [28]. Indandiones and their derivatives process important activity against Alzheimer’s disease and cancer. Donezepil for example acts as acetylcholineserase inhibitor for the treatment of mild (early stage) to moderate (middle stage) Alzheimer [29] whereas indanocine combat drug resistance malignancies [30]. Various research groups evaluated 2-arylidene-1,3-indandiones for cytotoxic properties [31,32]. It was later found that cytotoxicity and the fractional positive charge on the olefinic carbon atom could be increased by placing an additional electron-attracting oxygen atom into the indane scaffold [33]. 2-aryl-1,3-indandiones were found to present anti-inflammatory activities in a carrageenin edema test [34,35,36] 2-Phenyl-1,3-indandione appeared to be a weak inhibitor of prostaglandin biosynthesis [37]. However, the inhibition of prostaglandin biosynthesis by 2-aryl-1,3-indandiones showed no clear relationship with anti-inflammatory activity.

**Scheme 1:**
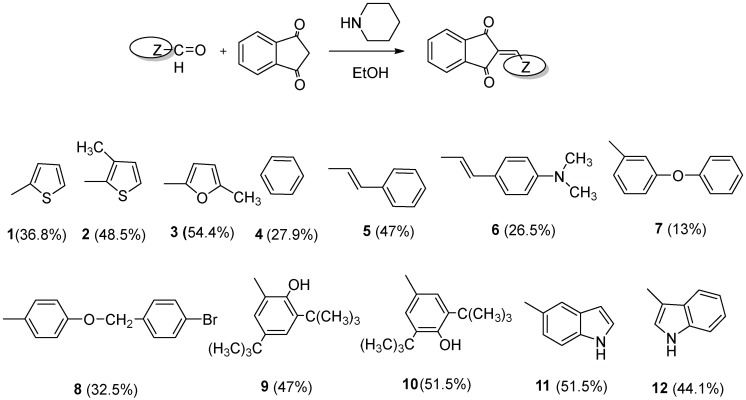
General synthesis of the new derivatives: 0.0068 mol of 1,3-indandione, 0.0075 mol of the corresponding arylaldehyde in 30 mL of absolute ethanol and piperidine 0.06 mL. Yields of the products are given in parentheses.

Thus, we decided to carry on our attempts to design and synthesize small biologically pleotropic effective compounds using the 1,3-indandionemoiety as an advantaged template for the design of multi-target agents. In our synthetic procedure several aromatic and heteroaromatic aldehydes properly substituted have been used in our effort to define the importance and correlation of steric and electronic parameters on the biological activities and to improve them through methodical alteration of the substituents. Thus, we present the synthesis and in vitro and in vivo biological evaluation of a series of 2-arylidene-1,3-indandiones (Scheme 1) diversely substituted.

Some of these derivatives, compounds **1**, **3**, **4**, **5**, **6**, **10** are referred in the literature as tyrosinase kinase inhibitors, in the treatment of schistosomiasis whereas **3** as a urease inhibitor. However their synthesis follow a different route from the described herein procedure [38,39,40,41,42,43,44,45,46,47,48,49,50,51].

## 2. Results and Discussion

### 2.1. Chemistry

The synthesis of the title compounds was accomplished via a Knoevenagel condensation reaction as presented in Scheme 1 as well as the structures of the various substituted aromatic aldehydes used for those derivatives. 2-aryl-1,3-indandiones were synthesized from aryl-aldehyde and 1,3-indandionein the presence of ethanol and piperidine as catalyst [33].

Compounds **2**, **3**, **5**, **9**, **10**, **11**, **12** were obtained in satisfactory yields (44–55%), compounds **1**, **4**, **6**, **8** in 25–40% yield while compound **7** was obtained in lower yield under our experimental synthetic procedure. The structure of the used aldehyde influences the yield of the reaction. It is known that Knoevenagel condensation reactions yields are influenced by the stereochemistry of the used aldehyde. Compounds **1, 3, 4, 5, 6, 10** have been synthesized earlier under different experimental conditions [38,39,40,41,42,43,44,45,46,47,48,49,50,51]. We used a different synthetic technique and the structures of the known compounds were verified according to the given in the literature spectral data, elemental analysis, or mps. In all cases our synthetic procedure was simpler (heating in alcohol with piperidine drops for eight hours) compared to the previous methods in which acidic conditions and heating for 21 h, microwave irradiation or specific catalysts were referred. 

Herein we synthesized them using our synthetic technique and we investigated their biological activities.

The final products were obtained by recrystallization from ethanol or ethanol/water or with preparative TLC with preparative TLC using CH_3_COOC_2_H_5_: petroleum ether (1:2), IR, ^1^H-NMR, ^13^C-NMR and elemental analysis were applied for the verification of the structures of the novel derivatives. LC/MS. The infrared spectra of the derivatives were determined. All the products displayed strong absorption for the carbonyl group indicating their stereo chemical homogeneity. The carbonyl absorptions were noted in the regions of 1680, 1700, 1720–1740 cm^−1^ in Nujol and 1625cm^−1^ for the C=C. The ^1^H-NMR spectra disclosed that they possessed the *Z* configuration of the olefinic protons. ^1^H-NMR spectroscopy indicated through integration the right analogy of aromatic and C*H*=protons. ^13^C-NMR spectra indicated the keto carbons (C=O) as well as the –C=C. The findings are in agreement with earlier publication [33]. The physicochemical properties of the novel derivatives are reported in the experimental session.

### 2.2. Physicochemical Studies

#### 2.2.1. Experimental Determination of Lipophilicity as R_M_ Values

Lipophilicity is a physicochemical parameter indicating the relationship of a drug to its biological activity. It affects ligand-target binding interactions, solubility, ADME (absorption, distribution, bioavailability, metabolism, and elimination) and toxicological effects. RPTLC (reverse phase thin layer chromatography) method was used for the experimental determination, as R_M_ values [52]. The RPTLC method is considered a secure, rapid and appropriate method for expressing the lipophilic character of a compound (Table 1). 

#### 2.2.2. In Silico Determination of Lipophilicity Values as Milog*P*

We used the Molinspiration program to calculate in silico the lipophilicity as milogP values. We tried to correlate the milog*P* values, the theoretically calculated lipophilicity in one equation with the R_M_ values of all the compounds (Table 2). However, this attempt, was found to be unsuccessful. Several factors, for example different solvation, silanophilic interaction, H-bridges, might cause this disagreement. It seems that a theoretically in silico calculated log*P* value is more accurate than an experimental [53].

#### 2.2.3. Molecular Properties Prediction-Lipinski “Rule of Five”

Traditional experimental procedures for the determination of human pharmacokinetic properties have been progressively replaced by the predicted since the computational tools that are in use are faster, simpler and more cost-effective [54]. Presently, a variety of use fulin silico methods have been developed for screening hundreds of data sets of compounds. In this regard, we obtained and entered in the online Molinspiration software version 2016.10 (www.molinspiration.com) [55] chemical structures and simplified molecular-input line-entry system (SMILES) notations of the synthesized indandiones to calculated various molecular properties., partition coefficient (log*P*), topological polar surface area (TPSA), hydrogen bond donors and acceptors, rotatable bonds, number of atoms, molecular weight, and to define the violations of Lipinski’s rule of five, in order to evaluate the drug-likeness of indandiones [56]. According to Lipinski’s rule poor absorption or permeation is more likely observed when there are more than 5 H-bond donors, 10 H-bond acceptors, the molecular weight (MW) is greater than 500 and the calculated Log*P* value is greater than 5. It is a rule of thumb to delineate if a chemical entity with a certain biological activity has drug-likeness, properties that would support its behavior as an orally active drug in humans. 

Log*P* values of all derivatives, as shown in Table 1, range from 2.52 to 5.94, except for **8**, **9**, and **10**. Since their lipophilicity was found to be less than 5, they did not violate “the rule of five” suggesting satisfactory permeability across cell membrane. LogBB is another important in silico descriptor to identify CNS active agents. Log*P* values were used for the theoretical calculation of the logBB values. For in silico prediction [57], compound with logBB value higher than 0.3 is considered to have high absorption through BBB whereas logBB values between 0.3 to −0.1 and lower than −0.1 are considered to be moderate and less absorbed through BBB. 

Log*P* values of 2-arylidene-1,3-indandionesand of the standard drug nordihydroguaretic acid (NDGA) were found to be under 5 defining their use *per os*. Furthermore, all derivatives were found to present MW less than 500. Thus, these compounds compared to large molecules, could be easily transported, diffused, and absorbed. The number of hydrogen bond acceptors (O and N atoms) and the number of hydrogen bond donors (NH and OH) in the synthesized compounds obey the Lipinski’s rule of five (less than 10 and 5 respectively). Within the data set compounds **1**–**7** and **11**, **12** seem to be orally active following Lipinski’s rule of five (Table 1).

TPSA is used as a significant indicator of the bioavailability of a bioactive molecule. This parameteris highly correlated with the hydrogen bonding properties of a molecule. TPSA of the derivatives was observed in the range of 34.14–54.37 A^0^. These values are well below the limit of 160A^0^ supporting the presence of good oral bioavailability. The upper limit for TPSA for a molecule to penetrate the brain is around 90 Å. The calculated results indicate that all structures are able cross BBB. 

### 2.3. Biological Evaluation

In this study, the novel derivatives were evaluated: (i) in vitro for their antioxidant profile and (ii) in vivo on the inhibition of the carrageenin-induced edema. They were also studied in vitro as possible inhibitors of soybean lipoxygenase, trypsin, and thrombin. 

The ability of the novel derivatives to present antioxidative ability in vitro was studied applying various biological protocols in which different radicals were generated. Factors such as solubility or steric hindrance are important and influence the experimental conditions. Two different methods were adopted: (i) the first measure the scavenging ability by donating a hydrogen- or an electron on a free radical used as anindicator of antioxidant activity and (ii) in the second a free radical is generated from an antioxidant system. The in vitro antioxidant activity was measured in terms of: (a) the interaction with the stable free radical DPPH; (b) the ABTS^+•^ radical cation reduction-decolorization ability, (c) scavenging of superoxide anion and (d) the anti-lipid peroxidation activity. 

The novel derivatives were studied for their interaction with the sTable 2,2-diphenyl-1-picrylhydrazyl free radical (DPPH)at concentration 50 µM, 100 µM and 200 µM after 20 and 60 min (Table 2) [58,59,60,61,62]. In this assay the DPPH radical is reduced transferring one electron from the antioxidant. Phenolic compounds such as catechol and derivatives generate phenoxide anions with strong antioxidant capacity. Nordihydroguaretic acid (NDGA), the reference compound acts by this mechanism. Compound **10** presents the highest scavenging activity at 100 µM after 20 min correlated with the presence of the phenolic OH. Compounds **3**, **4**, **5**, **7** presented medium activities depending from time and concentration. The presence of the heteroatom O or S seems to influence the activity e.g., derivatives **1**, **2**, and **3**. Replacement of phenyl ring (**4**) by a thienyl group (**1**) decreases antioxidant behavior. Both thienyl derivatives **1** and **2** exhibit very low activities. On the contrary the presence of the furyl ring (**3**) enhances reduction. Additionally, the presence of a π-dimethyl-amino group **6** reduces the scavenging ability whereas its absence in (**5**), increases the activity. The presence of two conjugated double bonds (**5**) leads to lower activity compared to the simpler compound **4.** Compounds **9** and **10** present the highest milog*P* values (5.94). Both include a phenolic hydrogen in their structure. However, compound **10** is the more active within the series since it generates more easily phenoxide anions. On the contrary in compound **9** steric reasons are possible to lead to a decrease. Lipophilicity seems to influence the interaction of compound **10**.

The radical cation ABTS^+•^ is directly generated through potassium persulfate by oxidation with no participation of an intermediary radical and then the reduction is followed by adding electron-donating antioxidants. In this assay the radical is generated before adding the antioxidant (decolorization assay). The compounds presented low to moderate antioxidant activity with most potent **10** and **7**. It seems that lipophilicity influences the activity, since both are presenting high lipophilicity values. To correlate the activity with the physicochemical parameters it was found that hydration energy as E_HYDR_ plays important role (Equation (1)).

log % ABTS^+•^ = 0.104 (± 0.036) E_HYDR_ + 2.175 (±0.366) (1)

*n* = 8, r = 0.945, r^2^ = 0.893, q^2^ = 0.762, s = 0.149, *F*_1,8_ = 5.356, α = 0.1

In the water soluble 2,2′-azobis(2-amidinopropane) hydrochloride (AAPH) the free radicals are produced in vitro through spontaneous thermal decomposition resembling to lipid peroxidation in cells due to the action of the peroxyl radicals. Among the synthesized derivatives compounds **1**, **2**, **9**, and **10** showed remarkable activity at final concentration 100 μΜ. Compounds **11**, **5**, **4** and **3** presented no activity under the experimental conditions. From the experimental results it can be concluded that the 3,5-di-tert-butyl-group offers protection against lipid peroxidation. Electronic parameters expressed by the highest occupied molecular orbital (HOMO) describes better the biological activity (Equation (2)).

log AAPH % = −22.042(± 8.731) HOMO − 4.853 (2.622)(2)

*n* = 8, r = 0.930, r^2^ = 0.864, q^2^ = 0.567, s = 0.146, *F*_1,8_ = 5.783, α = 0.1

The compounds have been tested to scavenge superoxide anion radicals. However, no results were recorded under the experimental conditions. Only **3** showed 20% scavenging activity at 100 μM.

Many researchers revealed the homology of soybean lipoxygenase to mammalian lipoxygenase [63,64]. Thus, we used soybean LOX in our in vitro experiments. Compound is **7** was found to be most potent followed by compounds **2**, **3**, **4**, **11**, **12**. Insertion of a methyl group (**2** > **1**) favors the inhibitory activity while replacement of a phenyl group from a thienyl does not seem to affect the activity (**1** and **4**). The heteroatom in the 5membered ring (furyl/thienyl) influences LOX inhibition (**2** > **3**). The presence of two conjugated double bonds (**5**) leads to lower activity compared to the simpler compound **4**. Indolyl derivatives in position 3- (**12**) present stronger inhibition than derivatives in 5-position (**11**). From the literature it has been found that lipophilicity is strongly associated with lipoxygenase inhibition [65]. However, in this series it seems that there is no correlation between lipophilicity and LOX inhibition. The combination of 1,3-indandione moiety with the double bond seems to be more significant feature and nodefinition is found for the importance of any other specific structural characteristics. Perusal of the antioxidant results as well as LOX inhibition did not support any correlation among these activities.

Serine proteases are implicated in the pathophysiology of inflammatory diseases and trypsin is well known years ago [66]. Trypsin has been associated with host defense reactions and tissue damage [67]. In this study, the novel derivatives have been evaluated towards trypsin and thrombin. Compounds **8** and **9** present high activity. On the contrary compounds **1**, **2**, **5**, **10**, and **12** do not present any activity under the experimental conditions. Thienylderivatives did not show any activity while the furyl-derivatives seem to be medium inhibitors.

It is well known that indandione derivatives exert their anticoagulant activity indirectly by inhibiting prothrombin synthesis in the liver [68]. It has been found that antioxidant compounds presenting antithrombotic effects play pivotal role in the therapy treatment of thrombosis based on vascular oxidative stress or inflammation [69]. Derivatives **1**, **2**, **5**, **8**, **9**, **10**, and **12** were tested in vitro as possible thrombin inhibitors. Among the tested derivatives compound **8** presented 100% inhibition followed by compounds **3** and **10**.

Carrageenin-induced rat paw edema (CPE) model was used to study the in vivo anti-inflammatory activity of the tested derivatives and the results are presented in Table 3. Compound **1** presented the highest anti-inflammatory activity from all the compounds, exhibiting even higher activity compared to the indomethacin used as reference compound. Compounds **2**, **7**, **8**, **10**, and **12** presented small differences in their biological activity. The presence of the thienyl group as well as of the furyl leads to higher anti-inflammatory activity (**1** and **3**) while substitution with a phenyl group leads to two-fold decrease of the activity (28%).

Correlation of the biological activity with the physicochemical parameters showed that lipophilicity as R_M_ values governs the activity (Equation (3)):

logCPE% = 0.349 (± 0.117)R_M_ + 1661(± 0.031) (3)

*n* = 6, r = 0.972, r^2^ = 0.945, q^2^ = 0.898, s = 0.022, *F*_1,6_ = 3.892, α = 0.1

### 2.4. Computational Studies—Docking Simulation Soybean Lipoxygenase

#### Molecular Modeling of the Synthesized Derivatives in Soybean LOX

**Figure 1:**
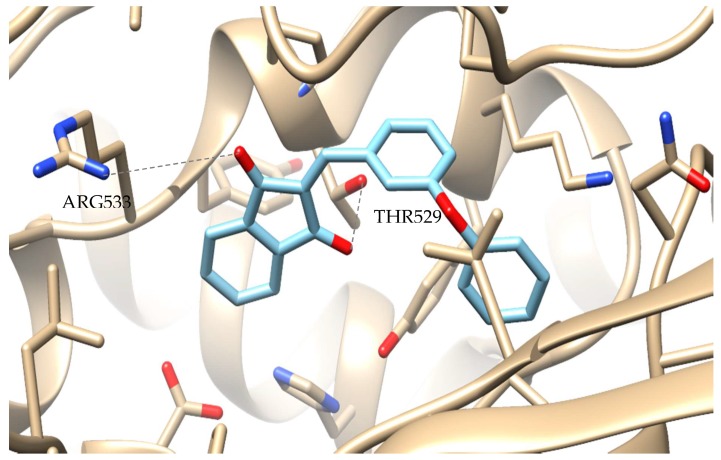
Preferred docking pose of 7(depicted in blue) bound to soybean lipoxygenase (LOX-1).

In silico docking has been performed for all the synthesized derivatives in accordance with the biological results. The favored docking position for the most promising indandione **7** is shown in Figure 1. Compound **7** has an AutoDockVina score of −10.2 binding to soybean LOX (PDB code: 3PZW). It is well known that the results from the in vitro inhibition of soybean lipoxygenase represent experimental values while docking scores are based on algorithms and scoring function calculations, so a 1 to 1 correlation is difficult to be reached. Docking describes the ligand binds to the protein. It seems that the novel compounds interact with the soybean lipoxygenase (SLOX) through allosteric interactions. Compound **7** present weak hydrogen bonds with ARG533 and THR529 with the two carbonyl groups of the 1*H*-indene-1,3(2*H*)-dione template and hydrophobic ones with the rest of the molecule. Since most LOX inhibitors act as antioxidants or by scavenging free radicals [70] and the oxidation of the enzyme occurs via a carbon-centered radical on a lipid chain, it is possible that compound **7** extendsinto the hydrophobic domain by blocking the substrates to the binding site and thus preventing oxidation.

## 3. Experimental Section

### 3.1. Materials and Instruments

All chemicals, solvents, chemical and biochemical reagents were of analytical grade and purchased from commercial sources (Merck, Fluka, and Sigma-Aldrich Laborchemikalien GmbH, Hannover, Germany and Alfa Aesar, Karlsruhe, Germany). Soybean lipoxygenase, albumin, trypsin, thrombin, sodium linoleate, 2,2′-azino-bis(3-ethylbenzothiazoline-6-sulfonic acid (ABTS), sodium linoleate, 2,2-azinobis-2-methyl-propanimidamine HCl (AAPH) were obtained from Sigma Chemical, Co. (St. Louis, MI, USA), 1,1-diphenyl-2-picrylhydrazyl (DPPH), nordihydroguairetic acid (NDGA), salycilic acid, warfarin, inogatran are purchased from the Aldrich Chemical Co. (Milwaukee, WI, USA). All starting materials were obtained from commercial sources (Merck, Merck KGaA, Darmstadt, Germany, Fluka Sigma-Aldrich Laborchemikalien GmbH, Hannover, Germany, Alfa Aesar, Karlsruhe, Germany, and Sigma, St. Louis, MO, USA) and used without further purification. 

Melting Points (uncorrected) were determined on a MEL-Temp II (Lab. Devices, Holliston, MA, USA). For the in vitro tests, Ultaviolet-Vis spectra were obtained on a Perkin–Elmer 554 double beam spectrophotometer (Perkin-Elmer Corporation Ltd., Lane Beaconsfield, Bucks, UK). Infrared spectra (film as Nujol mulls or KBr pellets) were recorded with Perkin–Elmer 597 spectrophotometer (Perkin–Elmer Corporation Ltd., Lane Beaconsfield, Bucks, UK). 

The ^1^H-NMR spectra were recorded at 300 MHz on a Bruker AM-300 spectrometer (Bruker AnalytischeMesstechnik GmbH, Rheinstetten, Germany) in CDCl_3_ or DMSO using tetramethylsilane as an internal standard unless otherwise stated. ^13^C-NMR spectra were obtained at 75.5 MHz on a Bruker AM-300 spectrometer in CDCl_3_ or DMSO solutions with tetramethylsilane as internal reference unless otherwise stated. Chemical shifts are expressed in _ (ppm) and coupling constants *J* in Hz. Mass spectra were determined on a LC-MS 2010 EV Shimadzu (Shimadzu, Kiyoto, Japan) using MeOH as solvent. Elemental analyses for C and H gave values acceptably close to the theoretical values (±0.4%) in a Perkin–Elmer 240B CHN analyzer (Perkin–Elmer Corporation Ltd., Lane Beaconsfield, Bucks, UK). Reactions were monitored by thin layer chromatography on 5554 F254 Silica gel/TLC cards (Merck and FlukaChemie GmbH Buchs, Steinheim, Switzerland). For preparative thin layer chromatography (prep TLC) Silica gel 60 F254, plates 2 mm, Merck KGaA ICH078057 were used. For the experimental determination of the lipophilicity using RPTLC TLC-Silica gel 60 F254 DC Kieselgel, Merck (20 × 20 cm) plates were used.

### 3.2. Chemistry General Procedure

In a round bottom flask 0.0068 mol 1,3-indandione and 0.0075 mol of the corresponding arylaldehyde were added and diluted in 30 mL of absolute ethanol using piperidine (0.06 mL) as catalyst. The mixture was heated reflux for approximately 8 h monitoring with TLC (petroleum ether/ethyl acetate, 7/3) for the completion of the reaction. The excess of the solvent was removed with rotary evaporator. The residue was filtered and purified either with recrystallization with EtOH 95°or ethanol/water or with preparative TLC using CH_3_COOC_2_H_5_: petroleum ether (1:2).

#### 3.2.1. (*Z*)-2-(thiophen-3-ylmethylene)-1*H*-indene-1,3(2*H*)-dione (**1**)

The precipitate was recrystallized from EtOH 95°. Yield: 36.8%; m.p. 176–177 °C; IR (Nujol) (cm^−1^): 1720, 1680, 1600, 730; ^1^H-NMR (300 MHz, CDCl_3_): δ (ppm) 7.78–7.81 (m, 2H, aromatics), 7.88 (d, 1H, aromatic, *J* = 5.5 Hz), 7.97–8.03 (m, 5H, aromatic and C=C*H*); ^13^C-NMR (75 MHz, CDCl_3_): 123.0, 123.1, 128.6, 134.9, 135.2, 136.3, 138.2, 141.7, 150.6, 173.6, 186.2, 187.3, 200.5, 203.5; Anal. C, H. Calcd %: (C14H8O2S) C: 69.98, H: 3.36 Found %: C: 70.4, H: 3.31 [38,39,40,41].

#### 3.2.2. (*Z*)-2-((3-methylthiophen-2-yl)methylene)-1*H*-indene-1,3(2*H*)-dione (**2**)

The residue was recrystallized from EtOH 95°. Yield: 48.5%; m.p. 171–172 °C; IR (Nujol) (cm^−1^): 1720, 1680, 730; ^1^H-NMR (300 MHz, CDCl_3_): *δ* (ppm) 2.60 (s, 3H, C*H*_3_), 7.07 (d, 1H, aromatic, *J*= 4.5 Hz), 7,77–7,98 (m, 5H, aromatics), 8,14 (s, 1H, C=C*H*); ^13^C-NMR (75 MHz, CDCl_3_): 15.4, 103.9, 122.9, 123, 131, 132, 134, 134.7, 135, 136.8, 140.3, 151.9, 161.1, 189.7, 216.1; Anal. C, H. Calcd %: (C_15_H_10_O_2_S) C: 70.84, H: 3.96 Found %: C: 70.98, H: 3.91.

#### 3.2.3. (*Z*)-2-((5-methylfuran-2-yl)methylene)-1*H*-indene-1,3(2*H*)-dione (**3**)

The precipitate was recrystallized from EtOH 95°. Yield: 54.4%; m.p. 130–136 °C; IR (Nujol) (cm^−1^): 1750, 1710, 1675; ^1^H-NMR (300 MHz, CDCl_3_): δ (ppm) 2.37–2.47 (m, 3H, CH_3_), 6.38-6.39 (br, 1H, aromatic), 7,69–7,95 (m, 6H, aromatics and C=C*H*), 8,56 (s, 1H, C=C*H*); ^13^C-NMR (75 MHz, CDCl_3_): 14.5, 112.2, 122.8, 129.1, 134.6, 142.3, 148.0, 155.7, 167.7, 180.2, 182.9, 186.0, 195.0, 199.8, 201.1; LC/MS (ESI): 237 [M]; Anal. C, H. Calcd %: (C_15_H_10_O_3_) C: 75.62, H: 4.23 Found%: C: 75.68, H: 4.50 [51].

#### 3.2.4. 2-benzylidene-1*H*-indene-1,3(2*H*)-dione (**4**)

The residue was recrystallized from EtOH 95°. Yield: 27.9%; m.p. 150–152 °C; IR (Nujol) (cm^−1^): 1730, 1680, 1580; ^1^H-NMR (300 MHz, CDCl_3_): δ (ppm) 7.53–7.55 (m, 4H, aromatics), 7.82–7.85 (m, 2H, aromatics), 7.92-8.02 (m, 3H, aromatics), 8.47 (d, 1H, C=C*H*, *J* = 7.3 Hz); ^13^C-NMR (75 MHz, CDCl_3_): 125.5, 126.0, 127.1, 127.3, 128.3, 128.5, 129.0, 130.2, 130.4, 134.0, 137.9, 138.2, 142.9, 193.3, 193.4 [42,43,44].

#### 3.2.5. (*Z*)-2-(3-phenylallylidene)-1*H*-indene-1,3(2*H*)-dione (**5**)

The residue was purified with preparative TLC using CH_3_COOC_2_H_5_: petroleum ether (1:2). Yield: 47%; m.p.165-167 °C; IR (Nujol) (cm^−1^): 1740, 1700, 1580; ^1^H-NMR (300 MHz, CDCl_3_): δ (ppm) 6.48–6.99 (m, 2H, C*H*=C*H*), 7.33–8.10 (br, 9H, aromatics), 9.31 (d, 1H, *J* = 8.4 Hz); ^13^C-NMR (75 MHz, CDCl_3_): 125.5, 125.8, 126.4, 126.5, 127.2, 127.3, 127.6, 127.8, 128.4, 128.5, 129.8, 137.0, 138.1, 138.2, 143.2, 144.0, 192.7, 192.8; Anal. C, H. Calcd %: (C_18_H_12_O_2_) C: 83.06, H: 4.65, Found %: C: 83, H: 4.60 [45,46,47].

#### 3.2.6. (*Z*)-2-(3-(4-(dimethylamino)phenyl)allylidene)-1*H*-indene-1,3(2*H*)-dione (**6**)

The residue was recrystallized from EtOH 95°/water. Yield: 26.5%; m.p. 85–87 °C; IR (Nujol) (cm^−1^): 1710, 1670, 1620; 1H-NMR (300 MHz, CDCl3): δ (ppm) 2.89–3.15 (m, 6H, N(CH3)2), 7.02 (d,1H, *J* = 8.24 Hz), 7.20 (d, 1H, *J* = 8.19 Hz), 7.35–7.90 (m, 8H, aromatics), 8.30 (d, 1H, J = 9.1 Hz); ^13^C-NMR (75MHz, CDCl_3_): 28.0, 39.9, 112.1, 112.3, 123.4, 125.6, 125.9, 127.2, 127.3, 128.3, 128.4, 131.0, 133.0, 136.0, 138.1, 138.2, 145.0, 151.2, 192.6, 192.7; Anal. C, H, N. Calcd %: (C_20_H_17_NO_2_) C: 79.19, H: 5.65, N: 4.62 Found %: C: 79.31, H: 5.28, N: 4.22 [48].

#### 3.2.7. (*Z*)-2-(3-phenoxybenzylidene)-1*H*-indene-1,3(2*H*)-dione (**7**)

The residue was recrystallized from EtOH 95°. Yield: 13%; m.p. 143–145 °C; IR (Nujol) (cm^−1^): 1730, 1680, 1620, 1150; ^1^H-NMR (300MHz, CDCl_3_): *δ* (ppm) 7.36–7.59 (m, 3H, aromatics), 7.81–8.22 (m, 5H, aromatics), 7.07–7.24 (m, 5H, aromatics), 9.96 (s, 1H, C=C*H*);^13^C-NMR (75 MHz, CDCl_3_): 114.0, 118.9, 119.0, 120.1, 124.5, 125.6, 125.8, 126.0, 127.2, 127.3, 129.5, 129.6, 130.5, 132.0, 135.3, 138.1, 138.2, 142.6, 154.4, 156.9, 192.9, 193.1; LC/MS (ESI): 327 [M + 1]; Anal. C, H. Calcd %: (C_22_H_14_O_3_) C: 80.97, H: 4.32, Found %: C: 81.29, H: 4.28.

#### 3.2.8. (*Z*)-2-(4-((4-bromobenzyl)oxy)benzylidene)-1*H*-indene-1,3(2*H*)-dione (**8**)

The residue was recrystallized from EtOH 95°. Yield: 32.45%; m.p. 222–224 °C; IR (Nujol) (cm^−1^): 1720, 1670, 1570, 1250; ^1^H-NMR (300 MHz, CDCl_3_): *δ* (ppm) 5.13 (s, 2H, OC*H*_2_), 7.06–7.08 (m, 2H, aromatics), 7.31–7.34 (m, 2H, aromatics), 7.53–7.56 (m, 2H, aromatics), 7.78–7.98 (m, 4H, aromatics), 7.99–8.00 (m, 2H, aromatics), 8.53–8.56 (m, 1H, C=C*H*); ^13^C-NMR (75 MHz, CDCl_3_): 70.5, 115.5, 115.6, 121.0, 125.7, 125.9, 127.3, 127.9, 127.9, 129.1, 129.3, 130.5, 131.5, 131.7, 131.9, 132.0, 134.3, 138.1, 138.2, 142.8, 159.4, 193.0, 193.2; LC/MS (ESI): 445 [M + K]^+^; Anal. C, H. Calcd %: (C_23_H_15_BrO_3_) C: 65.89, H: 3.61, Found %: C: 66.01, H: 3.58.

#### 3.2.9. (*Z*)-2-(3,5-di-tert-butyl-2-hydroxybenzylidene)-1*H*-indene-1,3(2*H*)-dione (**9**)

The product was recrystallized from EtOH 95°. Yield: 47%; m.p. 52–54 °C; IR (Nujol) (cm^−1^): 3500, 1750, 1710, 1600; ^1^H-NMR (300 MHz, CDCl_3_): *δ* (ppm) 1.12–1.64 (m,18H,2(C*H*_3_)_3_), 7.34–7.60 (m, 4H, aromatics), 9.86 (s, 1H, C=C*H*), 11.64 (s, 1H, O*H*); ^13^C-NMR (75 MHz, CDCl_3_): 29.2, 31.0, 31.3, 31.5, 34.0, 34.2, 35.0, 35.2, 119.9, 120.9, 127.8, 131.8, 132.0, 132.4, 136.0, 137.0, 137.6, 138.0, 140.7, 141.0, 141.6, 159.0, 197.0, 197.3; Anal. C, H. Calcd %: (C_24_H_26_O_3_) C: 79.53, H: 7.23, Found %: C: 79.89, H: 7.61.

#### 3.2.10. (*Z*)-2-(3,5-di-tert-butyl-4-hydroxybenzylidene)-1*H*-indene-1,3(2*H*)-dione (**10**)

The precipitate was recrystallized from EtOH 95°. Yield: 51.5%; m.p. 98–100 °C; IR (Nujol) (cm^−1^): 3350, 1710, 1680, 1625; ^1^H-NMR (300 MHz, CDCl_3_): δ (ppm) 1.35–1.78 (m, 18H, 2 × C(CH_3_)_3_), 7.73–8.52 (m, 5H, aromatics and *H*C=C*H*), 9.85 (s, 1H, O*H*); ^13^C-NMR (75 MHz, CDCl_3_): 30.4, 30.5, 34.0, 34.1, 124.8, 125.0, 125.7, 125.9, 127.1, 127.3, 127.6, 132.3, 136.0, 136.2, 138.1, 138.2, 142.6, 153.0, 193.1, 193.4.; Anal. C, H. Calcd %: (C_24_H_26_O_3_) C: 79.53, H: 7.23, Found %: C: 79.88, H: 7.55 [49,50].

#### 3.2.11. (*Z*)-2-((1H-indol-5-yl)methylene)-1*H*-indene-1,3(2*H*)-dione (**11**)

The residue was recrystallized from EtOH 95°. Yield: 51.5%; m.p. 223–225 °C; IR (Nujol) (cm^−1^): 3300, 1720, 1670, 1620; ^1^H-NMR (300 MHz, CDCl_3_): *δ* (ppm) 6.74 (s, 1H, aromatic), 7.29–7.30 (m, 1H, aromatic), 7.49–7.61 (m, 2H, aromatic), 7,78–7,80 (m, 2H, aromatic), 8.00–8,02 (m, 2H, aromatic), 8.47–8.50 (m, 2H, aromatic), 8.98 (s, 1H, C=C*H*); ^13^C-NMR (75 MHz, CDCl_3_): 100.8, 104.9, 105.9, 108.9, 111.0, 123.0, 129.0, 134.0, 134.9, 137.2, 144.0, 151.4, 158.5, 175.2, 180.6, 185.5, 186.6, 187.5; LC/MS (ESI): 313 [M+K]^+^; Anal. C, H, N. Calcd %: (C_18_H_11_NO_2_) C: 79.11, H: 4.06, N: 5.13 Found %: C: 79.22, H: 4.24, N: 5.32.

#### 3.2.12. (*Z*)-2-((1H-indol-3-yl)methylene)-1*H*-indene-1,3(2*H*)-dione (**12**)

The precipitate was recrystallized from EtOH 95°. Yield: 44.1%; m.p. 283–285 °C; IR (Nujol) (cm^−1^): 3250, 1720, 1650, 1590; ^1^H-NMR (300 MHz, CDCl_3_): *δ* (ppm) 6.89–6.99 (m, 1H), 7.38–8.03 (m, 8H aromatics), 8,35(s, 1H, C=C*H*), 9.8 (s, 1H, N*H*); ^13^C-NMR (75 MHz, CDCl_3_): 126.2, 127.0, 134.0, 137.0, 139.8, 146.0, 148.0, 150.8, 156.0, 157.7, 166.0, 167.2, 168.9, 174.0, 180.3, 187.7, 189.3, 204.4; LC/MS (ESI): 311 [M+K]^+^ (272); Anal. C, H, N. Calcd %: (C_18_H_11_NO_2_) C: 79.11, H: 4.06, N: 5.13 Found %: C: 79.16, H: 4.19, N: 5.04.

### 3.3. Physicochemical Studies

#### 3.3.1. Molecular Properties Prediction-Lipinski “Rule of Five”

Compounds were subjected to molecular properties prediction, drug-likeness by Molinspiration [55] (Table 1).

#### 3.3.2. Determination of R_M_Values

Reversed phase TLC (RPTLC) was performed on silica gel plates impregnated with 5% (*v*/*v*) liquid paraffin in light petroleum ether. The mobile phase was a methanol/water mixture (70/30, *v*/*v*). The plates were developed in closed chromatography tanks saturated with the mobile phase at 24 °C. Spots were detected under UV light. Five individual measurements of R_f_ values were recorded and used for the determination of R_M_ using the equation R_M_ = log [(1/R_f_) − 1] [59,60,62] (Table 2).

### 3.4. Biological Assays

#### 3.4.1. Biological In Vitro Assays 

Each in vitro experiment was performed at least in triplicate and the standard deviation of absorbance was less than 10% of the mean. For the in vitro assays, a stock solution (1% DMSO in the appropriate buffer with the tested compound diluted under sonication) was prepared from which several dilutions were made with the appropriate buffer.

##### Determination of the Reducing Activity of the Stable Radical 1,1-Diphenyl-picrylhydrazyl (DPPH) 

To a solution of DPPH in absolute ethanol an equal volume of the compounds with final concentrations of 50, 100 and 200 µM from a stock solution of 10 mM (in 1% DMSO and ethanol) was added. After 20 and 60 min the absorbance was recorded at 517 nm at room temperature [59,60,62] (Table 2).

##### ABTS^+•^—Decolorization Assay for Antioxidant Activity 

The radical cation ABTS^+•^ was generated from an ABTS stock solution in water (7 mM) which was mixed with potassium persulfate (2.45 mM) and left in the dark at room temperature for 12–16 h before use. The results are recorded after 1min of the mixing solutions at 734 nm and compared to the appropriate standard inhibitor Trolox [60] (Table 3).

##### Inhibition of Linoleic Acid Peroxidation

The oxidation reaction was started with 2,2′-Azobis(2-amidinopropane) dihydrochloride (AAPH) as a free radical initiator. Linoleic acid sodium salt was oxidized at 234 nm to a conjugated diene hydroperoxide resulting an increased absorption. Trolox was used as reference compound (93%) (Table 3).

##### Measurement of Superoxide Anion Radical Scavenging Activity

The superoxide anion producing system was set up by mixing PMS, NADPH, and air oxygen. The production of superoxide anion was estimated by the nitrobluetetrazole NBT method. The reaction mixture containing compounds, 3 μM PMS, 78 μM NADPH, and 25 μM NBT in 19 μM phosphate buffer pH 7.4 was incubated for 2 min at room temperature and the absorption was measured at 560 nm against a blank containing PMS. The tested compounds were pre-incubated for 2 min before adding NADPH. [59] Caffeic acid was used as an appropriate standard (76%).

##### Soybean Lipoxygenase Inhibition Study in Vitro 

In vitro study was performed as reported previously by our group [58,59,60,62]. Sodium linoleate (0.1 mM) and 0.2 mL of enzyme solution (1/9 × 104 *w/v* in saline) were used for the incubation of the tested derivatives. Sodium linoleate was converted to 13-hydroperoxylinoleic acid at 234 nm using NDGA as reference compound [58,59,60,62]. The results are given in Table 2.

##### In Vitro Inhibition of Trypsin Induced Proteolysis 

The Kunitz method was used after modification using bovine albumin was used as substrate for trypsin. In this assay, first 200 μL of 0.075 mg/mL trypsin and 780 μL of phosphate buffer pH 7.6 including the tested compounds (20 μL in DMSO, final concentration 100 μM) were pre-incubated at 37 °C for 20 min and then added 1 mL of albumin (stock solution 6 g/100 mL in phosphate buffer 0.1 M, pH 7.6) and incubated at 37 °C for 30 min. Secondly 1mLof 5% trichloroacetic acid was added to the incubated solution to stop the enzyme’s reaction and allowed to set at room temperature for 1 h. Then the solution was filtered, and the absorption of the filtrate was measured at 280 nm using salicylic acid as a reference compound [61] (Table 3).

##### In Vitro Inhibition of Thrombin 

As a substrate tosyl-Gly-Pro-Arg-pNA acetate salt was used at 1 mM final concentration. Compounds were dissolved at a final concentration of 0.1m in a Tris-buffer (0.05 M Tris, 0.154 M NaCl, ethanol 5%, pH = 8.0). Three minutes after the addition of bovine thrombin (2.5 unit/mg), the reaction was ended by adding 1 mL acetic acid 50%. The absorption of the released p-nitro-aniline was measured at 405 nm (Table 3) [71]. Warfarin and Inogatran were used as reference compounds.

#### 3.4.2. Biological in Vivo Assays

##### Inhibition of the Carrageenin-Induced Edema 

Edema was induced in the right hind paw of Fisher 344 rats (150–200 g) by the intradermal injection of 0.1 mL 2% carrageenin in water. Both sexes were used while females pregnant were excluded. Each group was composed of 6–15 animals. The animals, which have been bred in our laboratory, were housed under standard conditions and received a diet of commercial food pellets and water ad libitum during the maintenance but they were entirely fasted during the experiment period. Our studies were in accordance with recognised guidelines on animal experimentation. (guidelines for the care and use of laboratory animals published by the Greek Government 160/1991, based on EU regulations 86/609). Rats were kept in the Centre of the School of Veterinary Medicine (EL54 BIO42), Aristotle University of Thessaloniki, which is registered by the official state veterinary authorities (presidential degree 56/2013, in harmonization with the European Directive 2010/63/EEC). The experimental protocols were approved by the Animal Ethics Committee of the Prefecture of Central Macedonia (no. 270079/2500). The tested compounds 0.01 mmol/kg body weight were suspended in water, with a few drops of Tween 80 and ground in a mortar before use and were given intraperitoneally simultaneously with the carrageenin injection. The rats were euthanized 3.5 h after carrageenin injection. The difference between the weight of the injected and uninjected paws was calculated for each animal. The change in paw weight was compared with that in control animals (treated with water) and expressed as a percent inhibition of the edema CPE % values Table 2 [58,59]. Indomethacin in 0.01 mmol/kg (47%). Values CPE % are the mean from two different experiments with a standard error of the mean less than 10% (Table 3).

### 3.5. Computational Methods. Molecular Docking Studies on Soybean Lipoxygenase

UCSF Chimera was used for the visualization of the protein (PDB code: 3PZW) [72]. Water molecules were removed, missing residues were added with Modeler [73], hydrogen atoms and AMBER99SB-ILDN charges were added, and the charge on iron was set to +2.0, with no restraint applied to the iron atom and the ligands. Open Babel was used to generate and minimize ligand 3D coordinates using the MMFF94 force field [74]. Ligand topologies and parameters were generated by ACPYPE (AnteChamberPYthon Parser interfacE) [75] using Antechamber [76]. Energy minimizations were carried out using the AMBER99SB-ILDN force field [77] with GROMACS 4.6. Docking was performed with AutoDockVina (1.1.2) [78] applying a grid box of size 100 Å, 70 Å, 70 Åin X, Y, Z dimensions. The generation of docking input files and the analysis of the docking results was accomplished by UCSF Chimera. Docking was carried out with an exhaustiveness value of 10 and a maximum output of 20 docking modes. 

## 4. Conclusions

The designed and synthesized indandiones present multi-target activity against different targets such as inflammation, inhibition of enzymes, scavenging of free radicals, and inhibition of lipid peroxidation. In Table 4 the most significant indandiones in terms of activitiesare summarized.

In the DPPH assay the novel derivatives showed medium antioxidant activity with small differences dependent on time and concentration. The compounds moderately reduced at 100 μΜ the ABTS radical cation (ABTS^•+^) while they inhibited lipid peroxidation according to their structure. Compound **8** possessing the bromo-benzyloxy group seems to be the best trypsin and thrombin inhibitor. Compound **7** presents the better lipoxygenase inhibition while compound **1** the best anti-inflammatory activity in carrageenin rat paw edema. Thus, they can be considered to be lead compounds with multifunctional profile.

The pharmacokinetic prediction study offers useful results for the design and optimization of the ADME profile of the new derivatives. Thus, indandionesobeying Lipinski’s rule of five are expectedto be easily transported, diffused, absorbed being orally active with good oral bioavailability. All structures can cross BBB and act in CNS whereas the docking supports that the extension scaffold of compound **7** into the hydrophobic domain blocks approach of substrates to the active site and hence prevents oxidation by soybean LOX.

## Figures and Tables

**Table 1:** Molecular properties prediction-Lipinski “Rule of five”. Drug-likeness of the synthesized compounds.

Compd.	milog*P* ^a^	TPSA ^b^	N^o^ Atoms	N^o^ O,N ^c^	N^o^ OH, NH ^d^	N^o^ Violations	N^o^ Rotational Bonds ^e^	Volume ^f^	MW ^g^	logBBB ^h^
**1**	2.79	34.14	17	2	0	0	1	200.71	240.28	0.255
**2**	3.16	34.14	18	2	0	0	1	217.27	254.31	0.312
**3**	2.52	47.28	18	3	0	0	1	208.12	238.24	0.082
**4**	2.89	34.14	18	2	0	0	1	209.99	234.25	0.270
**5**	3.64	34.14	20	2	0	0	2	237.41	260.29	0.387
**6**	3.75	37.38	23	3	0	0	3	283.32	303.36	0.371
**7**	4.62	43.38	25	3	0	0	3	290.39	326.35	0.446
**8**	5.35	43.38	27	3	0	1	4	325.07	419.27	0.560
**9**	5.94	54.37	27	3	3	1	3	350.38	362.47	0.541
**10**	5.94	54.37	27	3	1	1	3	350.38	362.47	0.541
**11**	3.09	49.93	21	3	1	0	1	238.97	273.29	0.144
**12**	3.04	49.93	21	3	1	0	1	238.97	273.29	0.144

**N^o^** Atoms denotes the number of atoms in the compound; ^a^ Logarithm of partition coefficient between-octanol and water (milog*P*); ^b^ Topological polar surface area (TPSA); ^c^ Number of hydrogen bond acceptors (n-ON); ^d^ Number of hydrogen bond donors (n-OHNH); ^e^ Number of rotatable bonds (n-rotb); ^f^ Molecular Volume; ^g^ Molecular Weight; ^h^ blood–brain barrier (logBBB = 0.155 log*P* − 0.01TPSA + 0.164) [57].

**Table 2:** Lipophilicity values: experimental R_M_%. E*_HYDR_* Hydration Energy. E(HOMO). Interaction with the stable radical 1,1-diphenyl-picrylhydrazyl (DPPH), In vitro lipoxygenase (LOX) inhibitory activity at 100 µM.

Compd.	R_M_^a^ (±SD) ^b^	E*_HYDR_*	E_(HOMO)_	RA% 50 µM 20 min	RA% 50 µM 60 min	RA% 100 µM 20 min	RA% 100 µM 60 min	RA% 200 µM 20 min	RA% 200 µM 60 min	LOX (% Inhibition 100 µM)
**1**	0.031 ± 0	−20.48	−0.310777	2	0	16	16	2	6	32.6
**2**	0.031 ± 0	−17.37	−0.309938	2	2	16	16	4	7	45
**3**	0.014 ± 0	−7.67	0.291110	20	23	37	40	61	75	35.7
**4**	−0.615 ± 0.02	−6.53	−0.306356	5	10	26	32	23	32	35
**5**	0.158 ± 0	−6.71	−0.302600	21	25	44	48	57	72	26.4
**6**	−0.527 ± 0	−9.86	−0.271983	6	6	22	23	15	18	17.7
**7**	−0.196 ± 0.02	−7.30	−0.303773	27	35	40	50	75	85	67.3
**8**	*	−9.31	−0.314300	4	5	7	7	0	0	3.6
**9**	−1.038 ± 0	−5.68	−0.306356	4	3	3	1	6	6	no
**10**	−0.103 ± 0	−5.71	−0.314300	13	17	100	85	71	71	no
**11**	−0.196 ± 0.02	−12.71	−0.279993	2	2	19	19	10	10	38.6
**12**	−0.134 ± 0.02	−12.72	−0.279598	3	3	17	17	4	6	58.9
**NDGA**	-			81	83	87	93	94	96	93

^a^ R_M_ values are the average of at least 5 measurements, ^b^ SD standard deviation < 10%; * could not be measured under the reported experimental conditions.

**Table 3:** % Anti-lipid peroxidation (AAPH), Decolorization activity ABTS^+·^% assays. In vitro inhibition of trypsin induced proteolysis (% Trypsin Inh -Iptr%), thrombin inhibition TH%, In vivoanti-inflammatory activity (CPE %).

Compd.	AAPH% 100 µΜ	ABTS ^+.^ % 100 µΜ	Iptr% 100 µΜ	TH% 100 µΜ	CPE ^a^ %
**1**	90	9	no	no	57 **
**2**	92	2	no	-	45 *
**3**	no	20	17	39	50 *
**4**	no	19	4	no	28 *
**5**	no	no	no	-	-
**6**	9	22	29	19	-
**7**	82	37	33	no	41 **
**8**	79	16	85	100	46 *
**9**	96	no	98	0	-
**10**	85	39	no	27	42 *
**11**	no	8	6	-	-
**12**	31	no	no	-	42 **
**Trolox**	93	91			
**Indomethacin**					47
**Salycilic Acid**			54		
**Inogatran**				98	

* *p* < 0.01, ** *p* < 0.05. ^a^ % of reduction of the rat paw edema (CPE %) induced by carrageenin at the dose of 0.01 mmol/Kg/body weight.no: no action under the experimental conditions.

**Table 4:** Comparison table with multi-target pleiotropic compounds.

Compd.	% LOX Inh. 100 μM	% IPr 100 μΜ	% TH 100 μΜ	AAPH% 100 μM	% CPE
**7**	67.3			82	41
**8**		85	100	79	46
**9**		98		96	

## Abbreviations

AA: Arachidonic acid; AAPH: 2,2′-azinobis(2-amidinopropane) hydrochloride; ABTS: 2,2′-azino-bis(3-ethylbenzothiazoline-6-sulphonic acid; ADME: Absorption, distribution, bioavailability, metabolism and elimination; CHD: Coronary heart disease; Clog *P:*Theoretical calculated lipophilicity; COX: Cycloxygenase; CPE: Carrageenin-induced rat paw edema; CYP: Cytochrome P450; DPPH: 1,1-diphenyl-picrylhydrazyl; H(P)ETEs: 15-hydro(-pero)xy-eicosa-tetra-enoicacids; LOX: Lipoxygenase; LXs: Lipoxins; NADPH: reduced nicotinamide adenine dinucleotide phosphate; NDGA: Nordihydroguaretic acid; NBT: nitroblue tetrazolium; PGs: Prostaglandins; PMS: phenazine methosulfate; ROS: Reactive Oxygen Species; RPTLC: Reverse Phase Thin Layer Chromatography; SMILES: simplified molecular-input line-entry system; SLOX: soybean lipoxygenase; TCA: Trichloroacetic Acid.
